# Effectivines of drugs for insomnia treatment

**DOI:** 10.1192/j.eurpsy.2022.2100

**Published:** 2022-09-01

**Authors:** T. Jupe, B. Zenelaj

**Affiliations:** 1 Psychiatric Hospital of Attica, 5th Acute Psychiatric Department, Chaidari, Greece; 2 National Center for Children Treatment and Rehabilitationn, Child Psychiatry, Tirana, Albania

**Keywords:** sleep disorder, new drugs, Insomnia

## Abstract

**Introduction:**

Up to 10% of the US adult population will experience chronic insomnia, with women and elderly individuals at particularly high risk. Cognitive behavioral therapy is the core treatment for insomnia. When cognitive behavioral therapy is not enough, medications can help patients overcome the barriers and learned behaviors that prevent a good night’s sleep.

**Objectives:**

Through this research we aimed to investigate the effectiveness and safety of new drugs in the treatment of insomnia.

**Methods:**

We try to do a Bibliographic Review in PubMed using keywords like “insomnia” “new hypnotic drugs” and “effectiveness”

**Results:**

Patients receiving trazodone perceived better sleep quality than those receiving the placebo with a non-significantly moderate heterogeneity. As to secondary efficacy outcomes, we only found a significant reduction for trazodone in the number of awakenings compared to the placebo. Trazodone was effective in sleep maintenance by decreasing the number of early awakenings and it could significantly improve perceived sleep quality, although there were no significant improvements in sleep efficiency or other objective measures. Importantly, lemborexant improves latency to sleep onset and sleep maintenance and is able to help people who experience early morning awakenings. Safety data reveal that lemborexant has minimal residual effects on morning alertness or next day function.

**Conclusions:**

Unfortunately, treatment of insomnia is not always that simple. The disorder’s complex underlying pathophysiology warrants consideration of different nonpharmacologic and pharmacologic treatment options. Indeed, recent insights gained from research into the pathophysiology of insomnia have facilitated development of newer treatment approaches with more efficacious outcomes.

**Disclosure:**

No significant relationships.

